# The Skin Department: Its Object, Staffing, and Cost

**Published:** 1912-08-24

**Authors:** 


### THE IDEAL SCHOOL CLINIC.

### The Skin Department: Its Object, Staffing, and Cost.

Among the varieties of diseases particularly suitable for treatment at the school clinic are diseases of the skin. They are frequent among school children, and their treatment demands, in general, more time and attention than can be given by the parents. For that reason the treatment of such conditions at out-patient departments is generally unsatisfactory. The parents have no time to take the children regularly, and the simplest methods of treatment are often neglected at home to such an extent that the good done by weekly visits to the skin specialist at the hospital is evanescent. This is particularly the case with conditions that need care and attention, such as eczema and lichen. Serious conditions, such as tuberculosis of the skin, lupus, and confluent acne, can hardly be treated at the clinic, but the staff of the clinic will recognise its limitations, and send children suffering from these diseases to skin hospitals where they can obtain efficient treatment either as in- or out-patients. In these extreme cases the necessity for clinic treatment is less than in the minor degrees of skin disease. A child suffering from lupus, for instance, is not a fit child to be at school; he or she must be excluded from class, not because of risk of contamination to the other children, but simply because it is imperative that the treatment must be regularly and systematically carried out, and for that purpose it is necessary that the patient should be taken into hospital.

### The Diseases Treated.

The outfit required for the treatment of skin diseases at the school clinic is comparatively simple. No special room is needed; by proper arrangement of the time-table the surgeon's or physician's examining room may be placed at the disposal of the skin specialist for certain hours once every other day, or even every day, during the week. A couple of nurses, or one nurse at least, should be told off to attend to the skin cases. We have already laid stress on the importance of providing lavatory and bath accommodation at the clinic; the bath will be needed in most skin cases, and will be found a highly useful auxiliary. For the surgical treatment the instruments required will be found in the instrument cabinet; if excisions of small skin tumours are required, the case should be sent to the surgeon, who will also deal with such things as nævi and warts, except in cases where medical treatment is sufficient. Epilation is hardly ever necessary; as it is an operation requiring considerable skill and experience on the part of the surgeon, patients requiring it should be sent to a skin hospital and not treated at a clinic. Where an *x*-ray apparatus is kept, it will be found useful in some cases, but in general, with the exception of ringworm, cases requiring light treatment had far better be sent to a hospital than treated at the school clinic.

The diseases particularly suitable for treatment at the clinic are those that so frequently occur among school children. Scabies and parasitic skin diseases do well at the clinic, where they receive more individual attention than can be sometimes given at an overcrowded out-patient department of a general or special hospital. Eczema, particularly aural and facial eczema, chilblains, cracked hands and blistered feet, mild degrees of acne, particular skin diseases—among which pityriasis rosea stands first—are suitable for the clinic. The varieties of ringworm do especially well, whether treated by light or, in the case of circinata, by formaldehyde or iodine. Psoriasis and the lichens are often met with and are also suitable for clinic treatment. In fact the skin specialist will find that this department of the clinic can do excellent work at a relatively small cost.

We have referred twice to the "skin specialist" of the clinic. While it is, in our opinion, preferable that specialists should be attached to the clinic, we recognise that in this department at least the general practitioner is quite capable of dealing with the cases that come up for treatment. He meets these diseases constantly in general practice; he treats them, on the whole, admirably, even when he lacks the advantage of having a good nurse to aid him in carrying out the manipulation necessary to ensure cure. At the clinic he is therefore in a position to do good work. The only objection that may be made is that he is likely to overlook certain graver conditions that need bacteriological or microscopic examination to settle the diagnosis. But this is an objection that can hardly be pressed.

### The Cost of a Skin Department.

The outlay upon the skin department of the clinic will depend largely upon the percentage of serious cases treated. Taking an average of the usual class of prevailing school skin diseases—scabies and eczema—we venture to think that the cost per patient would not exceed sixpence. Where light treatment is required the cost will of course be considerably higher. Where many patients are treated for, say, ringworm, the cost may be brought down to a few shillings. To pay a guinea per patient for the efficient treatment of ringworm is to be wastefully extravagant. Where the physician is in the habit of ordering expensive drugs and ointments, the cost per patient will be naturally increased. But, as a rule, the cheaper ointments do excellently; the main thing is individual care and attention. Chrysarobin, ichthyol, and dermatol are rarely required, and a good physician will generally manage with their substitutes with equal benefit to the patient. Drugs for internal use are more expensive, unless stock mixtures are made up. This is a matter for the clinic dispenser, with whom the head of the skin department should co-operate.

It seems to us that the skin department is one of the most important and useful branches of the school clinic, indeed an indispensable branch. With it the clinic can do far more useful work than it would otherwise be capable of, and at a comparatively cheap rate.

